# Supplementary material for: Adsorption Characteristics of Bacterial Cellulose Membranes Toward Methylene Blue Dye in Aqueous Environment
Source: Gels. 2025 Sep 10;11(9):721. doi: 10.3390/gels11090721 (PMC12470013; doi:10.3390/gels11090721)
Supplement: Supplementary file 1 [file gels-11-00721-s001.zip › gels-3836435-supplementary.pdf]

gels

MDPI

# Adsorption Characteristics of Bacterial Cellulose Membranes toward Methylene Blue Dye in Aqueous Environment

## Supplementary Materials

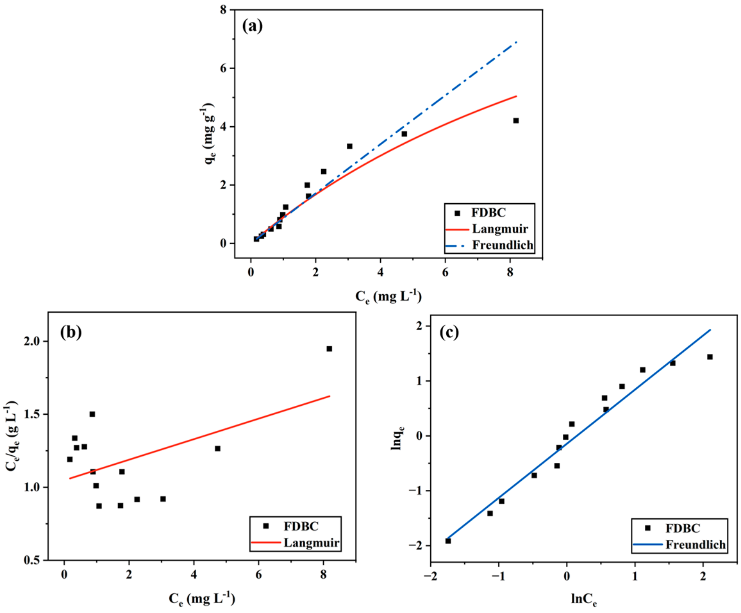


**Figure S1.** FDBC adsorption isotherms modeled by (a) non-linear Langmuir and Freundlich equations; (b) linear Langmuir equation; (c) linear Freundlich equation.

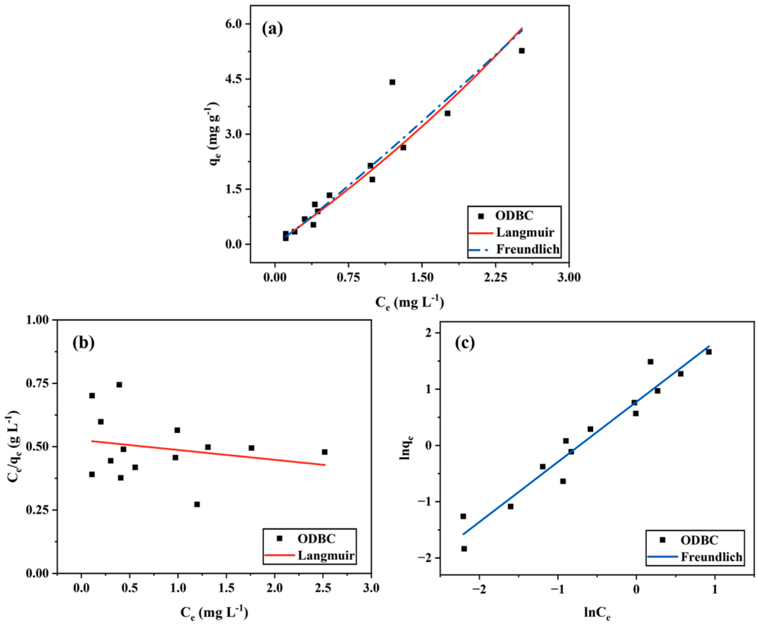


**Figure S2.** ODBC adsorption isotherms modeled by (a) non-linear Langmuir and Freundlich equations; (b) linear Langmuir equation; (c) linear Freundlich equation.

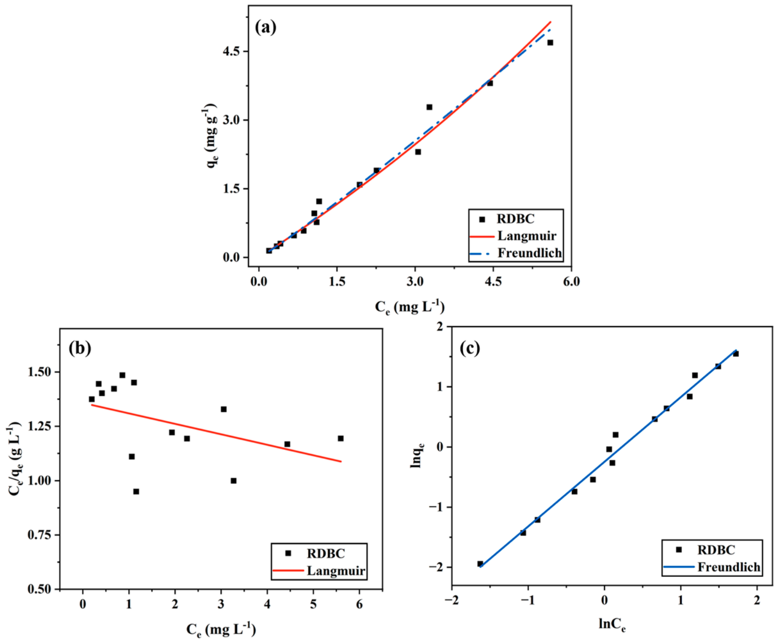


**Figure S3.** RDBC adsorption isotherms modeled by (a) non-linear Langmuir and Freundlich equations; (b) linear Langmuir equation; (c) linear Freundlich equation.

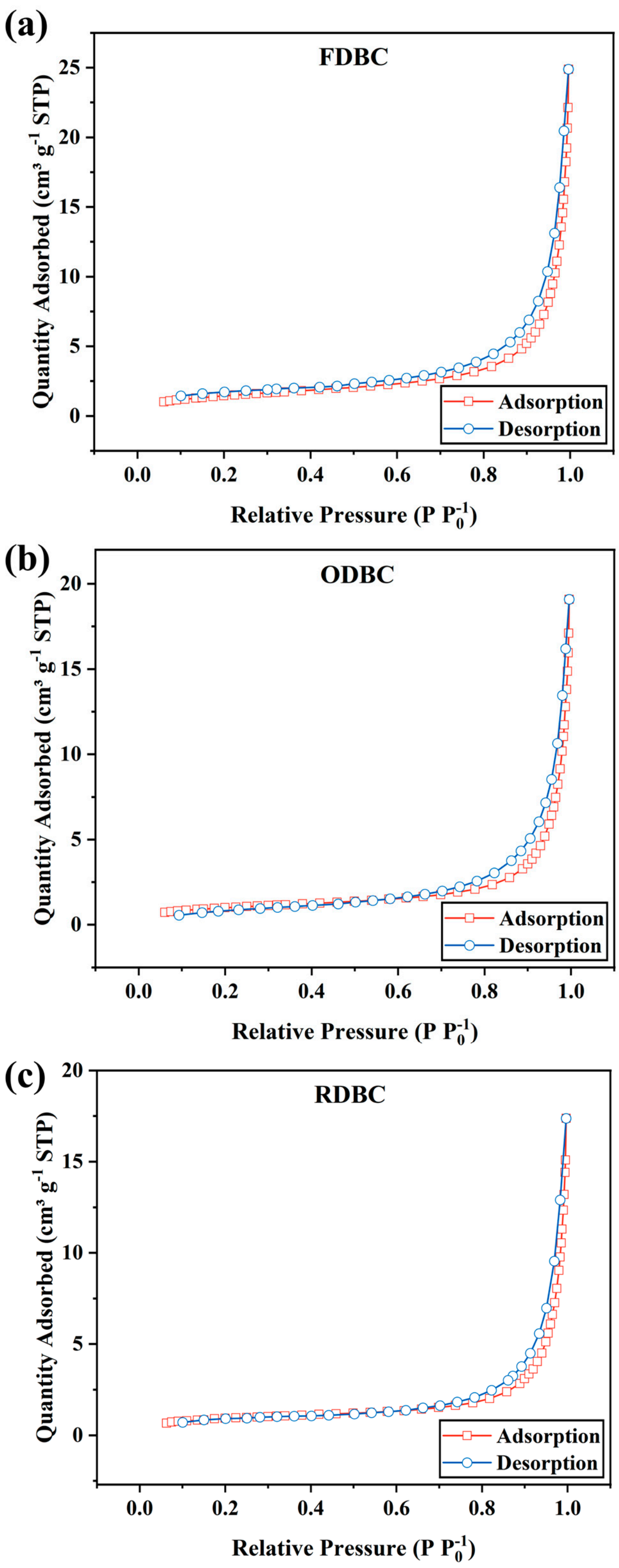


**Figure S4.** BET adsorption isotherm linear plot of (a) FDBC; (b) ODBC and (c) RDBC samples.

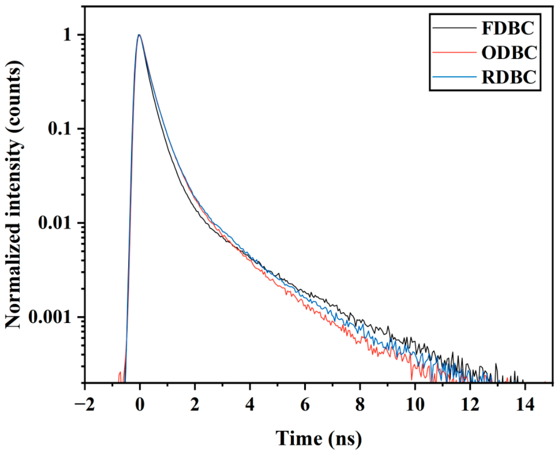


**Figure S5.** Background corrected and peak normalized PALS spectra of the BC samples.

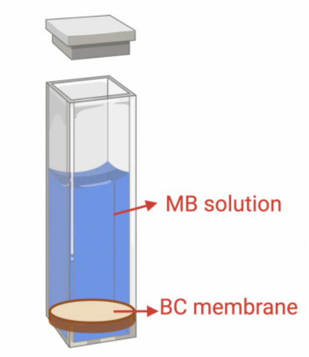


**Figure S6.** Schematic of the sample used in the kinetic tests.

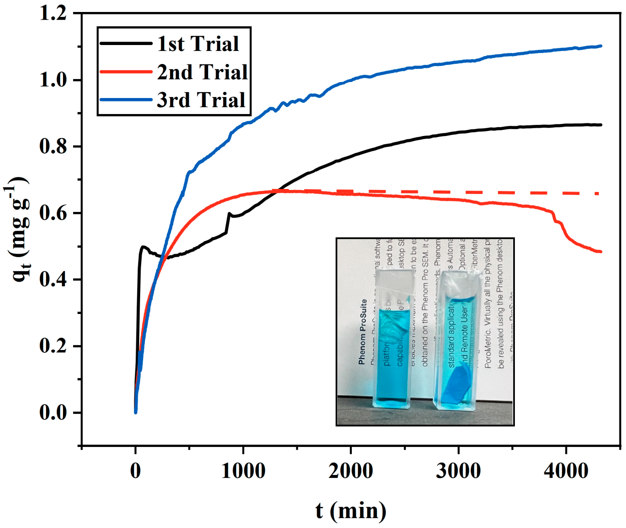


**Figure S7.** Effects of sample variations on FDBC adsorption tests.

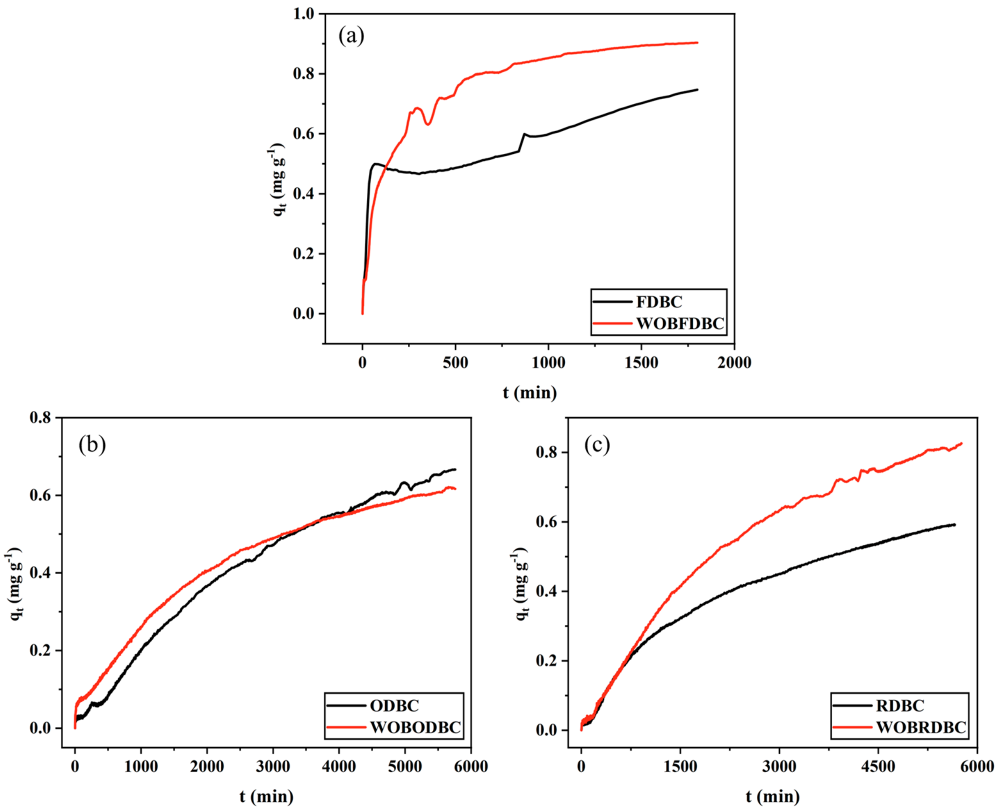


**Figure S8.** Effects of bleaching treatment on adsorptive behavior of (a) FDBC; (b) ODBC and (c) RDBC (WOB stands for "without bleach treatment").

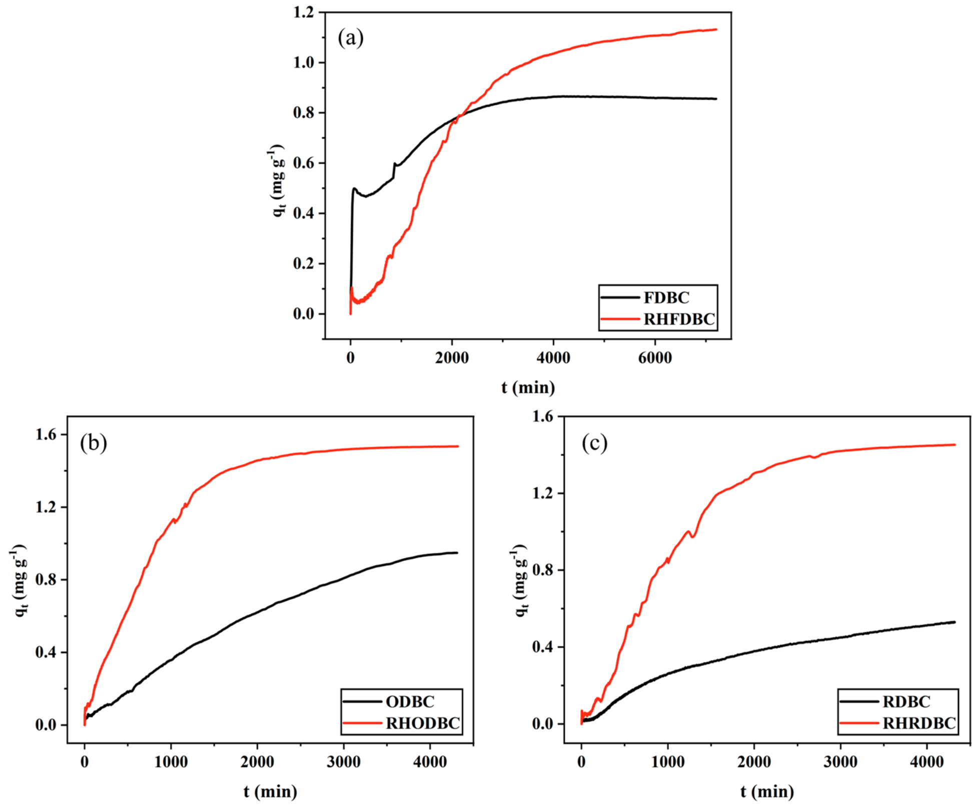


**Figure S9.** Effects of rehydration on adsorptive behavior of (a) FDBC; (b) ODBC and (c) RDBC.

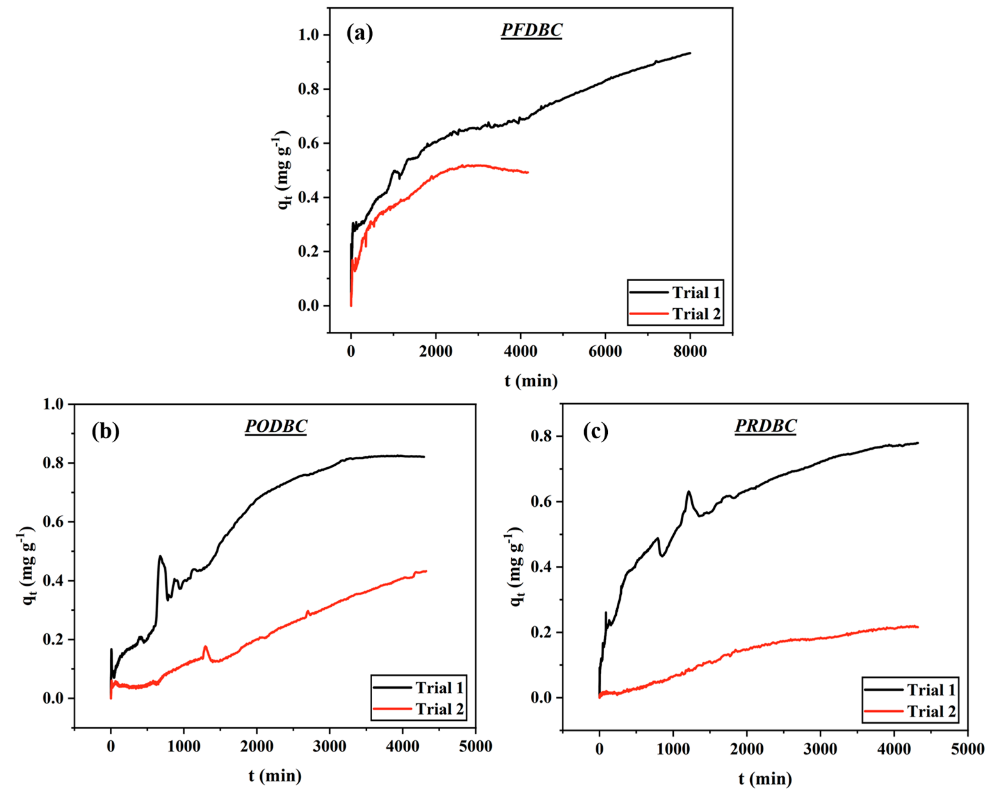


**Figure S10.** Adsorptive behavior of pure bacterial strain (*Novacetimonas hansenii*) produced (a) PFDBC; (b) PODBC; (c) PRDBC.

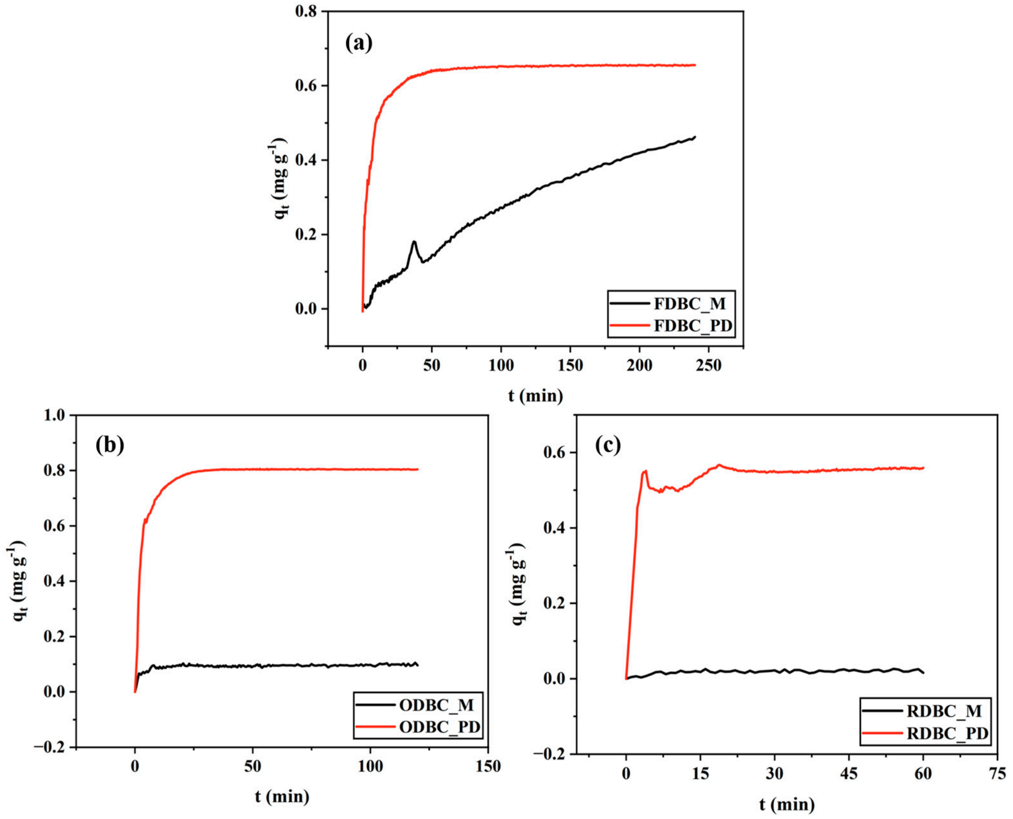


**Figure S11.** Adsorptive behavior of (a) FDBC; (b) ODBC; (c) RDBC powder samples (PD) versus membrane samples(M).

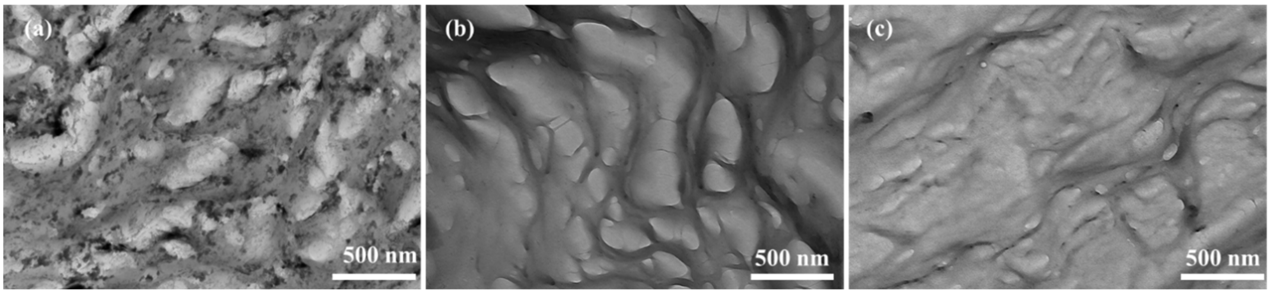


**Figure S12.** TEM images of the cross sections of (a) FDBC; (b) ODBC and (c) RDBC.

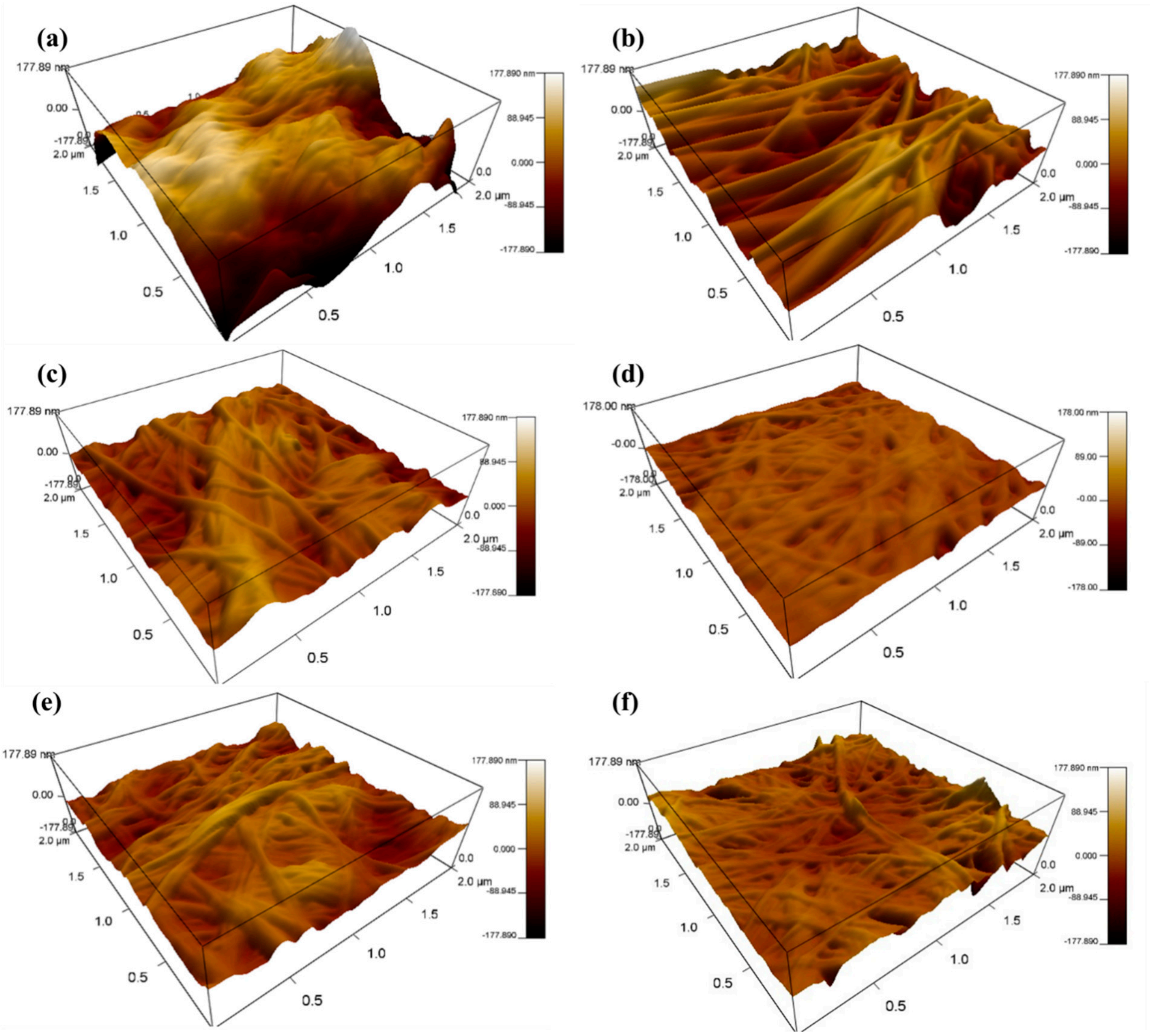


**Figure S13.** 3D AFM images of both sides of both sides of (a, b) FDBC; (c, d) ODBC; and (e, f) RDBC; (a, c, e) depict the surfaces exposed to air during the drying process, while (b, d, f) depict the surfaces that were in contact with the Petri dish or vacuum bottle.

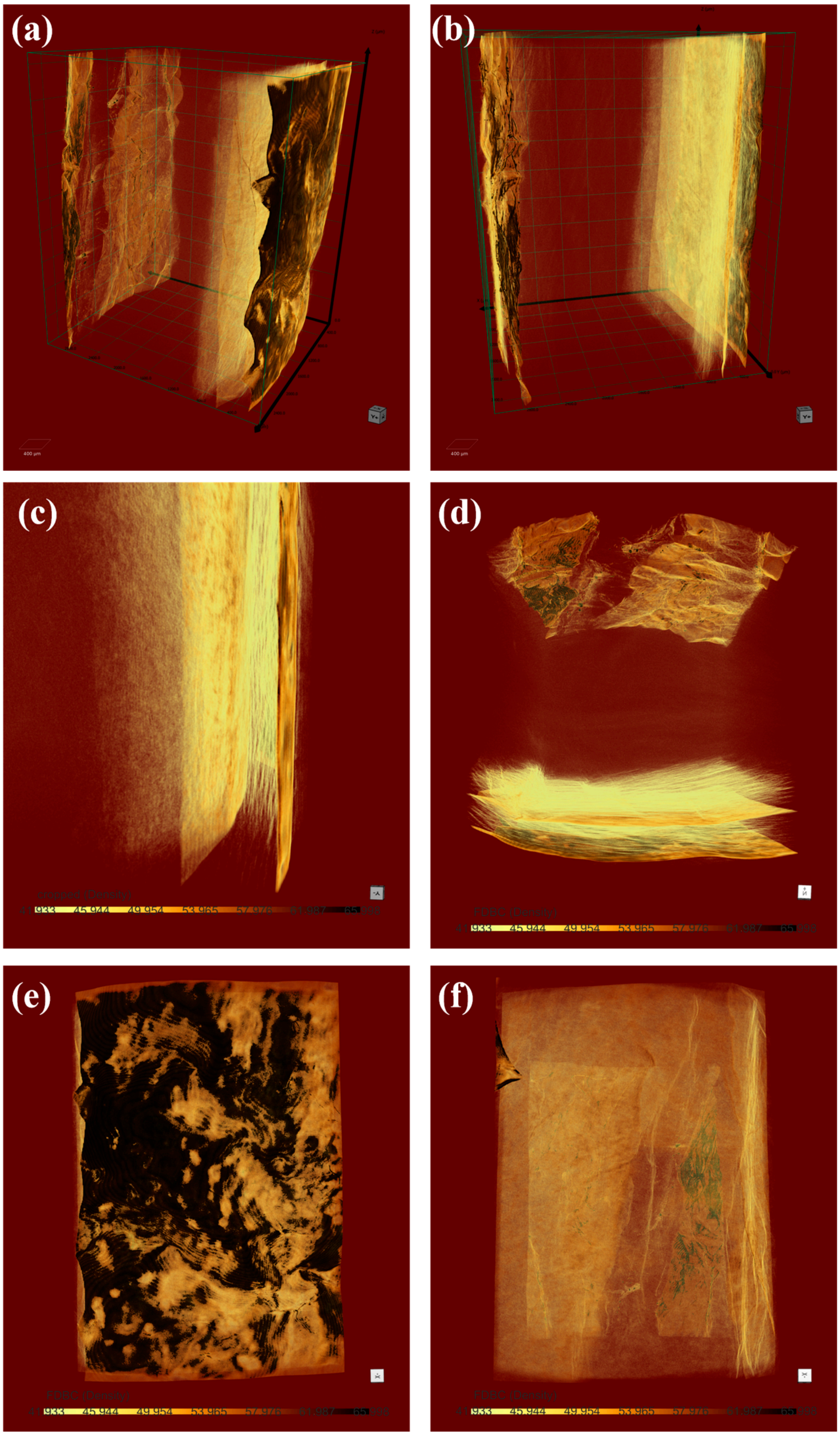


**Figure S14.** XRM images of an FDBC rectangular sample cut from a pristine FDBC membrane. The yellow regions represent cellulose fibrils; the hollow areas correspond to large voids where the interior was too porous to generate detectable signal. (a, b) Side views of the cross-sectioned surface created by cutting, showing the overall layered morphology, (c) magnified view of one side, highlighting the layered structure of FDBC, (d) side view focusing on the top-bottom arrangement of layers, (e) surface cross-sectional slice obtained from top-down scanning, showing the outer structure and (f) internal cross-sectional slice obtained from top-down scanning, revealing the inner fibrillar network.

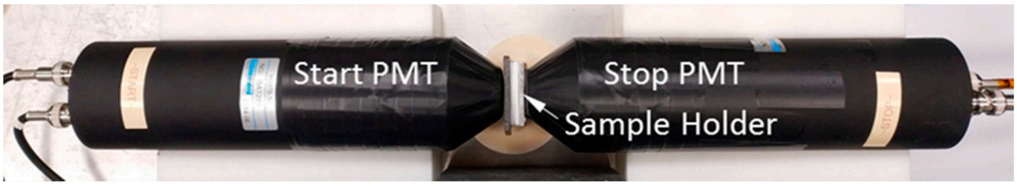


**Figure S15.** The Hamamatsu h3378-50 PMTs used in the PALS experiments as the start and stop detectors of the positron lifetimes. The sample sandwich is placed between the two PMTs.

**Table S1.** The fitted positron lifetimes and intensities from 4-lifetime free fittings.

| BC Type | $\tau_1$ (ps) | $I_1$ (%) | $\tau_2$ (ps) | $I_2$ (%) | $\tau_3$ (ns) | $I_3$ (%) | $\tau_4$ (ns) | $I_4$ (%) | $D_1$ (nm) | $D_2$ (nm) |
|---|---|---|---|---|---|---|---|---|---|---|
| FDBC | 181 | 33 | 391 | 55 | 1.55 | 7.1±2 | 3.41±0.18 | 5.0±4.1 | 0.481 | 0.781 |
| ODBC | 193 | 20 | 400 | 65 | 1.45 | 12.5±7 | 3.90±0.20 | 2.7±6.1 | 0.458 | 0.839 |
| RDBC | 206 | 24 | 409 | 62 | 1.42 | 12.1±7 | 3.90±0.24 | 2.1±5.4 | 0.451 | 0.839 |

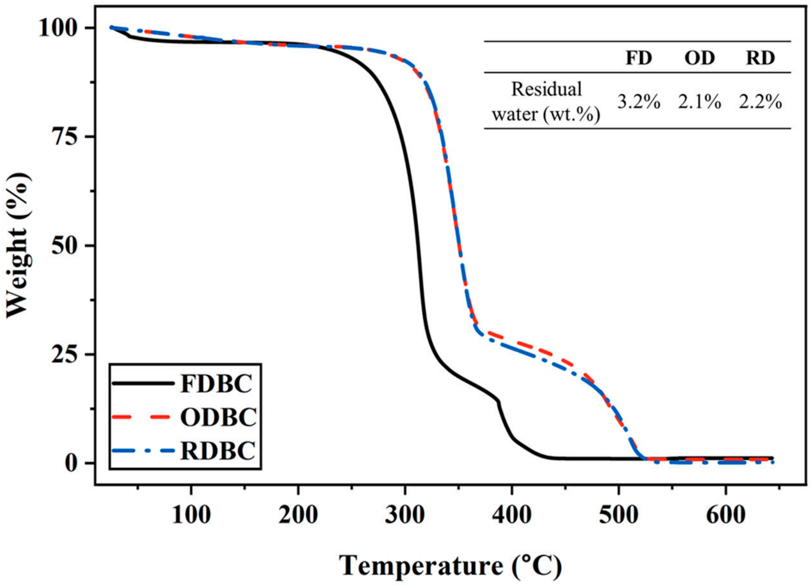


**Figure S16.** Comparison of TGA results among dried BC samples. Inset table indicates the residual water contents (wt. %) of every BC sample.

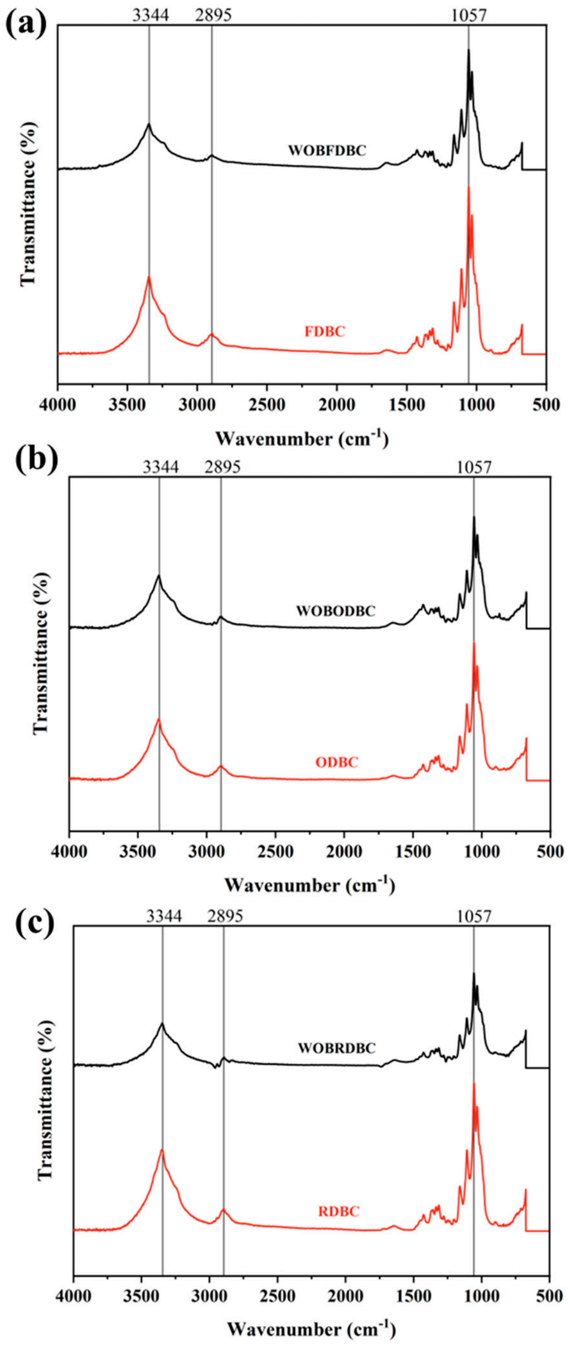


**Figure S17.** Comparison of FT-IR results between bleach-treated BC samples (red lines) and untreated BC samples (WOB, black lines). No significant differences in peaks were observed, indicating that the chemical structure of BC membranes remains unchanged by the bleach treatment.

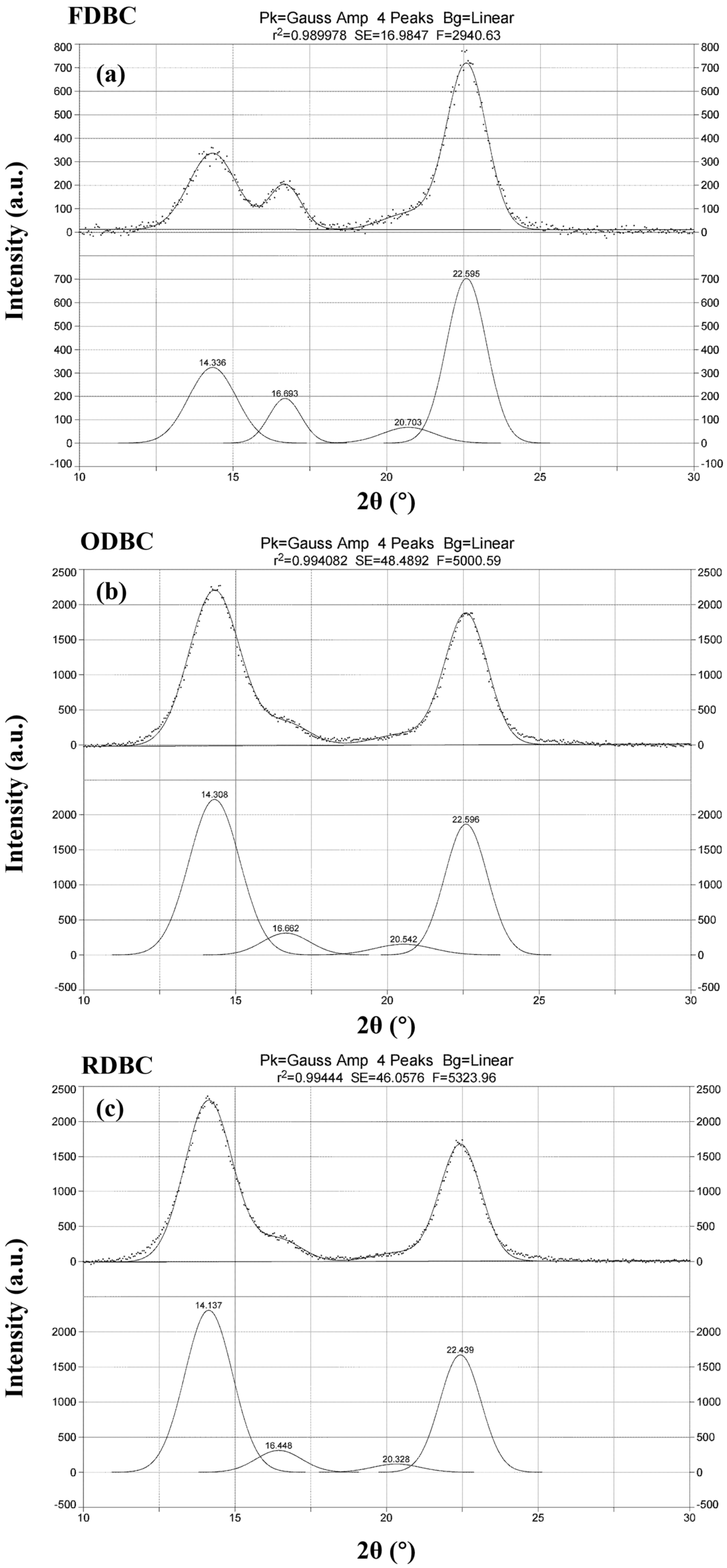


**Figure S18.** Peak deconvolution by means of PeakFit software and parameters are set as baseline model (linear, progressive Tol % Auto), peak type (spectroscopy and Guassian Amplitude) for (a) FDBC, (b) ODBC, and (c) RDBC.
